# Impact of Gastrointestinal Diseases on Health-Related Quality of Life of Patients in Pakistan

**DOI:** 10.7759/cureus.17374

**Published:** 2021-08-23

**Authors:** Hania Shahzad, Muhammad Muneeb Hussain, Adeel Abid, Saadia Sattar, Bisma Imtiaz, Shahab Abid

**Affiliations:** 1 Surgery, Aga Khan University, Karachi, PAK; 2 Medicine, Aga Khan University, Karachi, PAK; 3 Gastroenterology, Aga Khan University, Karachi, PAK

**Keywords:** quality of life, hrqol, gastrointestinal diseases, giqli, pakistan

## Abstract

Objective

This study aimed to evaluate the impact of gastrointestinal (GI) diseases on health-related quality of life (HRQoL) in Pakistani patients.

Methods

A cross-sectional study was conducted from 1st January 2019 to 15th February 2019 at a tertiary care hospital in Pakistan by employing a self-administered questionnaire called Gastrointestinal Quality of Life Index (GIQLI). Questionnaires were distributed among the patients presenting to outpatient gastroenterology clinics to evaluate their HRQoL.

Results

A total of 199 patients were included in this study, and their mean GIQLI score was 87.8 ± 17.8. Results showed that more severe core symptoms can lead to a poorer QoL. Overall GIQLI scores and most of its domain scores were lower in females as compared to males (p-value: 0.02). Obese patients had an overall lower mean GIQLI score including in the GI, psychological, and social domains while advanced age was associated with a higher disease-specific GIQLI score.

Conclusion

Based on our findings, GI diseases negatively impact the HRQoL in patients. Physicians need to consider the different domains of QoL as part of a holistic approach to treating these patients.

## Introduction

The prevalence of gastrointestinal (GI) symptoms is estimated to be high in patients presenting to outpatient clinics as well as among the general population [1]. Many GI disorders are characterized by chronic symptoms. A large population-based study conducted in the Netherlands estimated that the median duration of these symptoms is eight years [2]. Health-related quality of life (HRQoL) is an important parameter in modern medicine and refers to the extent to which an individual’s physical, emotional, and social well-being is affected by a medical condition and its treatment. Individuals with GI symptoms routinely report a lower HRQoL [3]. Studies have shown that patients with gastroesophageal reflux disease (GERD), irritable bowel syndrome (IBS), and ulcerative colitis report significantly more life stress and psychological distress than healthy patients. Furthermore, functional GI diseases lead to poor HRQoL, resulting in higher healthcare utilization [4,5]. Most studies describing the impact of GI diseases on HRQoL have largely focused on western populations [6].

The focus in healthcare has now predominantly shifted to improving the physical, psychological, and social well-being of individuals [7]. Failure to recognize and address these factors usually results in prolonged treatment courses and overutilization of healthcare resources [8].

Owing to the high prevalence of GI diseases in general, it has become evident that the burden of GI diseases on healthcare systems and their effect on the QoL needs to be seriously assessed. In this study, we aimed to determine 1) the mean Gastrointestinal Quality of Life Index (GIQLI) score of patients presenting to a tertiary care hospital in Pakistan, 2) the effect of GI diseases on HRQoL, and 3) factors associated with total GIQLI scores and individual domain scores.

## Materials and methods

Study design and setting

We conducted a cross-sectional study at a tertiary care university hospital in Karachi, Pakistan for a period of six months from August 2018 to February 2019. The ethical approval was obtained from the Ethical Review Committee (ERC) of the hospital.

Study population and sample size

Our study population included patients presenting to the gastroenterology outpatient clinic during the period specified above. A convenience sampling strategy was employed to recruit the participants for the study. A sample size of 192 patients was calculated with a mean GIQLI score [9] of 65 (±19) with a mean difference of 6 and a standardized effect size of 0.315. The power for calculation was kept at 80% with a two-sided confidence interval of 95%, adjusting for a 20% rate of attrition.

Eligibility criteria

All patients presenting to the gastroenterology outpatient clinic who were 18 years or older with a minimum body mass index (BMI) of 18 or above were included in the study. Furthermore, patients who had comorbid conditions such as diabetes mellitus, hypertension, ischemic heart disease, congestive heart failure, and cerebrovascular accidents were included. Patients with liver diseases and any other comorbidities not mentioned above were excluded from the study.

Data collection tool

Data was collected using a validated self-administered tool, i.e., GIQLI. It is an appropriate, validated, and potentially useful tool to assess HRQoL in clinical studies of patients with GI diseases and daily clinical practice [10] and can also be used on its own to assess the QoL of patients with GERD [6,11]. The questionnaire contains 36 items scored on a 5-point Likert scale (0-4 for individual questions and a range of 0-144 for total questions), where a higher score relates to better QoL. The five domains covered in the questionnaire are as follows: core GI symptoms, physical items, psychological items, social items, and disease-specific symptoms. The maximum score for physical, social, psychological, GI core symptoms, and disease-specific symptoms are 24, 16, 24, 40, and 40 respectively. GIQLI tool can differentiate between GI versus non-GI diseases; however, it cannot differentiate between various types of GI diseases [12]. Therefore, we did not attempt to sub-group the core symptoms according to specific diseases.

For the purpose of comprehensive analysis, we aimed to classify the overall GIQLI score and core symptom domain into two groups. Group A or group with excellent/good scores and Group B or group with fair/poor scores. The cut-off value was derived from the median of the maximum total score that can be achieved.

Overall GIQLI score

A total score of 144 gave us a median score of 72. Hence, Group A included participants with a total score greater than or equal to 72, and Group B included participants with a total score of less than 72. Groups A and B were a priori defined as excellent/good and poor/fair QoL groups.

Core symptom domain

There are 10 questions in the core symptoms domain with a minimum score of 0 and a maximum score of 4 for each question. A total of 10 questions gave us a maximum score of 40 that could be obtained in the core symptom domain, and a minimum of 0. Hence, the median value of 20 was considered the cut-off for Group A (>=20) and Group B (<20).

Study flow

Data collectors explained the study procedure in detail to the study participants along with the risks and benefits associated with participation in the study. Participation was voluntary and written informed consent was obtained from all participants. Multiple efforts were made to reduce bias, such as de-identification of forms by not including any personal identification details. Forms were marked by serial numbers for ease of data entry and the questionnaire was filled in private by the participants to reduce observation bias. All data obtained was kept confidential and was only accessible to the research team.

Data analysis

All analyses were conducted using Stata version 12.0 (StataCorp, College Station, TX). A descriptive analysis was performed, and results are presented as mean ± standard deviation or median [interquartile range (IQR)] for quantitative variables and number (percentage) for qualitative variables. The normality of all data sets was determined by using the Kolmogorov-Smirnov test. Total GIQLI scores and those for all four dimensions of the GIQLI were calculated. The Independent t-test or analysis of variance (ANOVA) was used to compare the total mean scores and those for dimensions with respect to age, gender, BMI, or comorbidities. Applied logistic regression was used to determine the factors associated with excellent/good and poor/fair GIQLI scores. All statistical tests were two-sided, and a p-value of 0.05 was considered statistically significant.

## Results

A total of 199 participants were enrolled in this study, out of which 115 (58%) were males and 84 (42%) were females. The mean age of males was 42.3 ± 13.4 years and that of females was 42.9 ±14.4 years. The mean BMI for males was 25.8 ± 4 kg/m^2^ and that for females was 25.3 ± 5.3 kg/m^2 ^(Table 1). The mean GIQLI score was 87.8 ± 17.8. The GIQLI consists of five domains. The mean scores for the five domains were as follows: GI symptoms: 22.5 ± 6.4, physical activity: 12.4 ± 5.4, psychological domain: 11.6 ± 4.6, social activity domain: 10.2 ± 3.1, and the disease-specific domain: 31.1 ± 6.2 (Table 2).

**Table 1:** Characteristics of the study population (n=199) BMI: body mass index; SD: standard deviation

Variables	Female (n=84)	Male (n=115)
Age in years, mean ± SD	42.9 ± 14.4	42.3 ± 13.4
≤50, n (%)	57 (67.9)	80 (79.6)
>50, n (%)	27 (32.1)	35 (30.4)
Height, mean ± SD	1.6 ± 0.1	3.2 ± 15.9
Weight, kg, mean ± SD	63.9 ± 11.3	73.6 ± 13.2
BMI, kg/m^2^, mean ± SD	25.3 ± 5.3	25.8 ± 4.7
Underweight, n (%)	1 (1.2)	1 (0.9)
Overweight, n (%)	21 (25.0)	39 (33.9)
Obese, n (%)	17 (20.2)	24 (20.9)
Comorbidities, n (%)	26 (30.9)	32 (27.8)
Diabetes mellitus, n (%)	12 (14.3)	8 (7.0)
Hypertension, n (%)	18 (21.4)	22 (19.1)
Cerebrovascular accident, n (%)	0 (0.0)	1 (0.9)
Ischemic heart disease, n (%)	2 (2.4)	1 (0.9)
Congestive heart failure, n (%)	0 (0.0)	2 (1.0)
Others, n (%)	3 (3.6)	4 (3.5)

**Table 2:** Total quality of life and domain scores (n=199) CI: confidence interval; GI: gastrointestinal; GIQLI: Gastrointestinal Quality of Life Index; IQR: interquartile range; SD: standard deviation

GIQLI domains	Mean ± SD	95% CI	Median (IQR)	Min-max
Overall	87.8 ± 17.8	85.3–90.3	87 (75–101)	45–130
GI symptoms	22.5 ± 6.4	21.6–23.4	23 (18–26)	3–38
Physical	12.4 ± 5.4	11.7–13.2	13 (9–24)	0–24
Psychological	11.6 ± 4.6	10.9–12.2	12 (8–15)	2–20
Social	10.2 ± 3.1	9.7–10.6	10 (8–12)	2–16
Disease-specific	31.1 ± 6.2	30.2–31.9	32 (27–36)	13–40

Patients with GI diseases had low scores in six items of symptom domain, including blood in the stool, trouble with uncontrolled stool, trouble with swallowing, urgent bowel movement, and trouble with diarrhea. Low scores were observed in three items of physical function, namely waking up at night, feeling unfit, loss of endurance, and change in appearance.

Overall, the GIQLI score was significantly lower in females as compared to males (84.4 ± 16.4 vs. 90.3 ± 18.5; p-value: 0.02). Physical (11.1 ± 5.4 vs. 13.4 ± 5.3), psychological (10.8 ± 4.4 vs. 12.2 ± 4.6), and disease-specific (30.1 ± 6.5 vs. 31.8 ± 5.8) domain scores were lower in females as compared to males, and these differences were statistically significant (p-values: 0.003, 0.03, and 0.05 respectively).

Individual domains of GIQLI were analyzed with respect to age brackets of <30, 31-40, 41-50, and >50 years. The figures showed an upward trend in the disease-specific domain of GIQLI scores, which increased as the age bracket increased as follows: 29.3 ± 6.5, 30.7 ± 6.6, 31.9 ± 5.9, and 32 ± 5.3 respectively.

Analysis of the BMI of patients revealed that patients in the obese-level BMI category had an overall lower mean GIQLI score than patients in the normal BMI category (86.9 ± 17 vs. 89.1 ± 8.4) including GI (21.8 ± 6.9 vs. 23.3 ± 6.5), psychological (10.9 ± 4.1 vs. 12.1 ± 4.4), and social domains (9.8 ± 2.7 vs. 10.3 ± 3.1).

With respect to comorbidities, patients without any comorbidities had higher physical (12.9 ± 5.1 vs. 11.4 ± 6.2) and psychological scores (11.9 ± 4.6 vs. 10.9 ± 4.4) than patients with comorbidities: p-values of 0.09 and 0.19 respectively.

All other GIQLI domains were found to be statistically significant when compared with GI symptom scores (Table 3). Scores for Group 1 and Group 2 (overall GIQLI scores of <72 and >72) were analyzed using applied logistic regression for factors predicting excellent/good and poor/fair overall GIQLI score, and they showed no significant association with respect to age, gender, height, BMI, weight, and comorbidity. With a one-year increase in age, the odds of excellent/good GIQLI score were 1.02 [95% CI: 0.99-1.05].

**Table 3:** Factors predicting a low gastrointestinal score CI: confidence interval; SD: standard deviation

Domains	Gastrointestinal score			
	<20, mean ± SD (n=68)	≥20, ± SD (n=131)	Odds ratio [95% CI]	P-value
Physical activity	10.1 ± 5.1	13.7 ± 5.2	0.89 [0.82–0.97]	0.008
Psychological	9.9 ± 4.1	12.4 ± 4.6	0.87 [0.78–0.97]	0.008
Social	9.1 ± 2.9	10.7 ± 3.0	0.77 [0.65–0.91]	0.001
Disease-specific	29.4 ± 6.6	31.9 ± 5.7	0.87 [0.80–0.94]	<0.001

## Discussion

Our study showed that patients with more severe GI core symptoms have a poorer QoL. Overall, female gender, obesity, and older age (>50 years) were associated with a lower score across various individual domains. However, age, gender, height, BMI, weight, and comorbidity did not significantly predict good/excellent or fair/poor overall GIQLI scores.

Our results revealed that core symptoms had the strongest association with the HRQoL. Our findings are consistent with the existing literature, which has shown that other GI diseases including functional GI disorders, inflammatory bowel diseases, and IBS adversely affect the HRQoL. Furthermore, their increased severity has been linked with poorer HRQoL scores [12,13]. Indeed, GI diseases are not a major cause of mortality; however, more severe disease often translates into chronic pain, fatigue, and psychological distress [14,15]. Our findings strengthen the need for emphasizing symptomatic management and improving the QoL for patients with GI diseases. Gastroenterologists should focus their efforts on normalizing the lives of their patients. Physicians should identify red flags for psychological distress so that early interventions can be made. Specialized training for gastroenterology providers that incorporates psychological principles such as positive psychology inpatient care should be explored [16].

Our findings showed that men reported an overall higher HRQoL and better scores in every domain when compared to females. This is inconsistent with a study conducted in Pakistan evaluating the impact of GERD on QoL where no significant difference was found [5]. However, our study used GIQLI, instead of the GERD impact score, which is more comprehensive since it covers both upper and lower GI symptoms as compared to the GERD impact score. Additionally, females more frequently report GI symptoms compared to men [2]. It could be attributed to the fact that men generally refrain from reporting symptoms [17].

Patients of older age reported a lesser impact of disease-specific symptoms on their HRQoL. This is consistent with the literature where older age (>65 years) was considered a positive predictor for higher HRQoL [2]. This finding can be attributed to the superior quality of care given to older family members' disease symptoms in the South Asian population. A significant correlation has been seen between the degree of obesity and the change in GIQLI scores [18,19]. Total GIQLI score and subscores have even been shown to improve 24 hours after bariatric surgery [20]. Obese people in this study were similarly found to have lower mean overall GIQLI scores and lower mean scores in GI symptoms as well as psychological and social domains. However, this difference between BMI categories in relation to different GIQLI domains was not statistically significant. This could be due to the fact that there were considerably fewer obese subjects in our cohort compared to normal-weight individuals, and even fewer morbidly obese patients.

Multimorbidity is negatively associated with HRQoL [21], and certain disease combinations, such as diabetes and coronary disorders, have a more severe impact on HRQoL than others. In a study determining the prevalence of GI symptoms and their effect on the HRQoL among patients with diabetes mellitus, a negative correlation was observed between GIQLI score and GI symptoms among diabetics [22]. In the study, while not being statistically significant, people with comorbidities had a lower mean score in psychological and physical domains as compared to people with no comorbidities. This could be explained by the fact that comorbidities are widely prevalent in the Pakistani population and hence do not have a profound impact on the perception of one’s QoL.

Although higher GIQLI scores correlate with better HRQoL, the authors of the questionnaire had not created distinct high or low score ranges. In this study, we created an arbitrary range by dividing the scores into half, which may correlate with good or poor HRQoL. We used multivariate analysis to identify a factor that would predict the score in a particular range. With our cut-offs, age, BMI, or comorbidities are not associated with either of the categories (i.e, excellent/good or fair/poor). To the best of our knowledge, this is the first attempt in the literature to categorize the score ranges. Our study proves that this arbitrary division does not effectively correlate with good or bad HRQoL. Other methods need to be explored to categorize these cut-off values.

Physicians need to consider the different domains of HRQoL as part of holistic care. This needs to be incorporated into training guidelines and enforced by hospital administrators, especially in the developing world. Patients with lower HRQoL should be identified earlier in their course of treatment. Moreover, in terms of further research, similar studies stratifying good and poor HRQoL scores should be conducted in this region to determine whether age, gender, BMI, or comorbidities could be independent factors in determining the level of HRQoL being experienced during GI illness.

The major strength of this study is the use of a structured and validated tool, i.e., GIQLI, which increases the reliability and reproducibility of our results [23]. Equipped by the strength of this tool, we have used data in this study to conduct a comprehensive multivariate analysis across various domains and subgroups to extensively ascertain the impact on HRQoL.

The results of our study were based on data from a single hospital. However, patients presenting to our hospital represent a wide and diverse array of study subjects that provides a representative sample of the country's population. Additionally, we did not have data on patients with GI diseases who did not visit clinics, which might give a falsely lower GIQLI score. However, we assume that those patients would not have significant impairment in their functionality as they would have sought help if they did.

## Conclusions

Based on our findings, the severity of the GI disease can lead to poor QoL outcomes in the domains of physical, social, and psychological well-being. In particular, the presence of severe GI core symptoms leads to poorer outcomes in all the other domains representing the QoL. Considerable gender differences were found with respect to the overall GIQLI scores and scores for physical and psychological domains, with females generally manifesting lower scores compared to males.
